# MARIJUANA USE AMONG OLDER ADULTS: HARMS MAY OUTWEIGH BENEFITS

**DOI:** 10.1093/geroni/igz038.740

**Published:** 2019-11-08

**Authors:** Namkee G Choi, Diana M DiNitto, Stephan Arndt

**Affiliations:** 1 University of Texas at Austin, Austin, Texas, United States; 2 University of Iowa, Iowa City, Iowa, United States

## Abstract

The US Epidemiologic data show that past-year marijuana use rates among the 50+ age group rose more than 300% between 2002 and 2017. In 2017, 10.2% of those aged 50-64 years and 3.7% of those aged 65+ years were past-year users, and 15% of these users reported all or part of their use to have been for medical purposes. In this symposium, using findings from previous research and recent epidemiologic data, we will present both positive and adverse effects of late-life marijuana use. Marijuana may be useful for the treatment of pain (particularly neuropathic pain) and chemotherapy-induced nausea and vomiting. However, older marijuana users are often long-term users and significant proportions suffer from marijuana use disorder along with other substance (nicotine, alcohol, prescription and illicit drugs) use and/or mental disorders. Adverse effects also include dizziness, confusion, impairments in memory and attention, somnolence, and hallucinations, which may outweigh purported positive effects.

